# Investigation of the Role That NADH Peroxidase Plays in Oxidative Stress Survival in Group B *Streptococcus*

**DOI:** 10.3389/fmicb.2018.02786

**Published:** 2018-11-20

**Authors:** Michelle L. Korir, Rebecca A. Flaherty, Lisa M. Rogers, Jennifer A. Gaddy, David M. Aronoff, Shannon D. Manning

**Affiliations:** ^1^Department of Microbiology and Molecular Genetics, Michigan State University, East Lansing, MI, United States; ^2^Division of Infectious Diseases, Department of Medicine, Vanderbilt University Medical Center, Nashville, TN, United States; ^3^Tennessee Valley Healthcare Systems, Department of Veterans Affairs, Nashville, TN, United States; ^4^Department of Pathology, Microbiology, and Immunology, Vanderbilt University Medical Center, Nashville, TN, United States; ^5^Department of Obstetrics and Gynecology, Vanderbilt University Medical Center, Nashville, TN, United States

**Keywords:** group B *Streptococcus*, *Streptococcus agalactiae*, macrophages, oxidative stress, intracellular survival

## Abstract

Macrophages play an important role in defending the host against infections by engulfing pathogens and containing them inside the phagosome, which consists of a harsh microbicidal environment. However, many pathogens have developed mechanisms to survive inside macrophages despite this challenge. Group B *Streptococcus* (GBS), a leading cause of sepsis and meningitis in neonates, is one such pathogen that survives inside macrophages by withstanding phagosomal stress. Although a few key intracellular survival factors have been identified, the mechanisms by which GBS detoxifies the phagosome are poorly defined. Transcriptional analysis during survival inside macrophages revealed strong upregulation of a putative NADH peroxidase (*npx*) at 1 and 24 h post-infection. A deletion mutant of *npx* (Δ*npx*) was more susceptible to killing by a complex *in vitro* model of multiple phagosomal biochemical/oxidant stressors or by hydrogen peroxide alone. Moreover, compared to an isogenic wild type GBS strain, the Δ*npx* strain demonstrated impaired survival inside human macrophages and a reduced capacity to blunt macrophage reactive oxygen species (ROS) production. It is therefore likely that Npx plays a role in survival against ROS production in the macrophage. A more thorough understanding of how GBS evades the immune system through survival inside macrophages will aid in development of new therapeutic measures.

## Introduction

Group B *Streptococcus* (GBS; *Streptococcus agalactiae*) is well-known for its ability to cause adverse pregnancy outcomes and severe neonatal infections. Moreover, GBS can cause infections in the elderly as well as immunocompromised individuals (Johri et al., 2006). In neonatal infections, GBS can ascend the maternal vaginal tract, cross the extraplacental membranes and cause an infection *in utero*. Alternatively, the baby can become infected while passing through the birth canal by inhaling GBS when present in vaginal secretions (Katz and Bowes, 1988; Doran and Nizet, 2004). Despite the use of prevention strategies like intrapartum antibiotic prophylaxis, which have been successful in reducing the incidence of neonatal infections overall, the current rates of neonatal infections have plateaued over the years, indicating the need for alternative therapies and preventative measures (Verani et al., 2010; Russell et al., 2017).

Macrophages play an important role in defending the host from invading pathogens. In the pregnant uterus, both maternally-derived decidual macrophages and fetally-derived placental macrophages play roles in maintaining maternal immune tolerance to the fetus while helping defend against pathogens that may infect and traverse the extraplacental (fetal) membranes to infect the baby *in utero* (Heikkinen et al., 2003; Petroff et al., 2003; Kim et al., 2012). Upon recognition by a macrophage, a pathogen is typically phagocytosed and contained inside a phagosome that, through a maturation process, becomes highly microbicidal, consisting of reactive oxygen and nitrogen species, antimicrobial peptides, heavy metals, low nutrients, and acidic pH (Flannagan et al., 2012). Previous studies have reported that GBS is capable of withstanding this extreme environment and can persist inside the fully mature phagolysosome for an extended period of time without inhibiting phagosome maturation (Valentin-weigand et al., 1996; Cornacchione et al., 1998; Cumley et al., 2012; Korir et al., 2016). This ability to survive inside macrophages may explain its ability to cross host cell barriers, effectively evade the immune system, and persistently colonize women, even after antibiotic treatment.

An important aspect of the highly microbicidal environment GBS endures inside the macrophage is the production of reactive oxygen species (ROS). ROS can also be produced as a by product during metabolism and represent a source of endogenous ROS; therefore many bacteria require ways to inactivate or detoxify ROS (Cabiscol et al., 2000). The amount of ROS produced by the NOX2 NADPH oxidase in macrophages during the ROS burst, however, is significantly higher, inducing oxidative stress to kill invading pathogens (Slauch, 2011). ROS can target and damage lipids, proteins, DNA, and RNA, making it particularly harmful to the cell (Cabiscol et al., 2000). Despite being catalase negative, GBS has been shown to survive exposure to high levels of hydrogen peroxide (H_2_O_2_), a type of ROS (Poyart et al., 2001; Cumley et al., 2012; Korir et al., 2016). Moreover, the ability to withstand a combination of phagosomal stressors, including H_2_O_2_, varied among strains of different sequence types (STs). Specifically, strains belonging to ST-17, considered to be a hypervirulent lineage, were better able to survive compared to strains of other lineages (Korir et al., 2016). One mechanism that GBS uses to defend against ROS is superoxide dismutase, which aids in detoxification of superoxide by converting it to O_2_ and H_2_O_2_ (Poyart et al., 2001); however, the mechanism by which GBS detoxifies H_2_O_2_ has yet to be examined. GBS also produces glutathione as well as a carotenoid pigment, both of which have been shown to protect against oxidative damage (Liu et al., 2004; Janowiak and Griffith, 2005).

In this study, we sought to address the extent to which a putative NADH peroxidase, encoded by *npx*, fosters GBS survival within the macrophage and the ability to withstand ROS. These studies demonstrate that expression of *npx* is highly upregulated during survival inside macrophages and that Npx plays a role in defending against oxidative stress and is important for reducing ROS production inside macrophages.

## Materials and Methods

### Bacterial Strains and Growth Conditions

The GBS strain used in this study was GB00112, a strain isolated from a vaginal rectal screen of a woman who had recently given birth (Manning et al., 2008) and was classified as sequence type (ST)-17 using multilocus sequence typing. This strain has previously been examined by our group for host cell attachment and intracellular survival in macrophages (Korir et al., 2014, 2016). Isogenic mutants of this strain, including a *npx* deletion mutant (Δ*npx*) harboring the empty vector (GB02168), a complemented *npx* mutant harboring a plasmid containing the *npx* locus, referred to as Δ*npx*:*npx* (GB02169), and the parental strain harboring the empty vector alone (GB02139) also were used in this study. Bacterial strains were grown on Todd-Hewitt agar (THA) plates or in Todd-Hewitt broth (THB) at 37°C. Derivatives harboring the pLZ12 plasmid were grown in media supplemented with 3 μg/mL chloramphenicol. *E. coli* DH5α strains used for the mutation and complementation process were grown in LB broth or agar supplemented with either 150 μg/mL erythromycin or 20 μg/mL chloramphenicol when necessary.

### Cell Culture

THP-1 monocyte-like cells (ATCC TIB-202) were cultured in RPMI medium with 2 mM L-glutamine (Gibco), 10% fetal bovine serum (FBS; Atlanta Biologicals), and 1% penicillin/streptomycin (Gibco) at 37°C with 5% carbon dioxide. THP-1 cells were differentiated into macrophages by incubation with 100 nM phorbol 12-myristate 13-acetate (PMA; Sigma) in RPMI medium with 2% FBS for 24 h as described (Schwende et al., 1996).

### Macrophage Survival Assays

Intracellular survival assays were described previously (Korir et al., 2016). Briefly, GBS strains were grown to mid-log phase in THB. PMA treated THP-1 macrophages were then infected with GBS strains at a multiplicity of infection (MOI) of 10:1 in RPMI for 1 h. Extracellular bacteria were killed by first washing any unattached bacteria and adding RPMI supplemented with 2% FBS, 100 μg/ml gentamicin (Gibco), and 5 μg/ml penicillin G (Sigma) to the wells. At the indicated time points, intracellular bacteria were enumerated by lysing the macrophages with 0.1% Triton-X (Sigma), and serially diluting and plating lysates to calculate CFUs. Survival was normalized to the total amount of bacteria present in the well after the 1 h infection period (final inoculum). In a separate series of experiments, the macrophages were treated with 500 μM apocynin or 0.1% DMSO as a negative control for 30 min prior to infection with GBS to inhibit production of ROS; the macrophages remained in the medium containing apocynin or DMSO for the duration of the experiment. The number of bacteria surviving intracellularly with apocynin treatment was normalized to survival in DMSO.

### Generation of GBS Competent Cells and Construction of Isogenic Bacterial Mutant Strains

Group B *Streptococcus* electrocompetent cells were generated as previously described with modification (Framson et al., 1997). GBS was grown in THB with 0.5 M sucrose and a sublethal, but inhibitory concentration of glycine to early log phase (OD = 0.25) in shaking conditions. The culture was then pelleted at 4°C, the supernatant removed, and the pellet washed with ice cold 0.625 M sucrose solution. The culture was pelleted again and resuspended in 0.625 M sucrose and stored at -80°C.

The *npx* gene was deleted using the thermosensitive plasmid pG^+^host5 as previously described (Al Safadi et al., 2011; Parker et al., 2017). The 5′ and 3′ flanking regions of *npx* were amplified using the primer sets *npx-*P1/*npx-*P2 for 5′ and *npx-*P3/*npx-*P4 for 3′ regions (Table 1). The two amplified flanking regions were combined in a crossover PCR, resulting in a single PCR product of the deletion sequence. The PCR product and the pG^+^host5 plasmid were digested with *BamH*I and *Kpn*I then ligated to create pG^+^host5Δ*npx*. This plasmid was electroporated into GB112 competent cells and transformants were selected by growth on 2 μg/ml erythromycin at 28°C. Cells in which the plasmid integrated into the chromosome via homologous recombination were selected for by growth on erythromycin at 42°C. Colonies that grew at 42°C were then grown in broth without antibiotic selection at 28°C for several passages to allow for excision of the plasmid. The cultures were then plated and single colonies were tested for erythromycin susceptibility and screened for gene deletion using PCR with primers *npx-*P5 and *npx-*P6. Deletion was confirmed by sequencing.

**Table 1:** Oligonucleotide primers used to generate GBS mutagenesis and complementation vectors.

Primer name	Sequence (5′ to 3′)
*npx*-P1^a^	CCGC**GGATCC**CCAAAGCCCCAGATTTTGCTG
*npx*-P2^b^	CCCATCCACTAAACTTAACAAAGAGGATTGGGCTATGCGA
*npx*-P3^b^	TGTTTAAGTTTAGTGGATGGGACCATAAAGTCGGTCTCAGCA
*npx*-P4^c^	GGG**GGTACC**TTAGACCCCATTATGAGGCTGC
*npx*-P5	CTAAGGCTGCGTCTAACCGT
*npx*-P6	TGCTGTAGCTATTGCAGCGT
*npx-*pLZ12-F2^a^	CGC**GGAT**CCAGGAGGACAGCTATGACAGAAAAATATGTA
*npx-*pLZ12-R2^d^	AAAA**CTGCAG**CTATTCGCATAGCCCAATCC


*^a^BamHI restriction site is shown in bold. ^b^Complementary sequences for P2 and P3 sets are underlined. ^c^KpnI restriction site is shown in bold. ^d^PstI restriction site is shown in bold.*

Complementation of the *npx* deletion was done using the pLZ12 plasmid under control of the *rofA* constitutive promoter (Neely et al., 2003). The coding sequence of *npx* was amplified using the *npx-*pLZ12 primer set (Table 1). The PCR product and the pLZ12-rofApro plasmid were digested with *BamH*I and *Pst*I and ligated to create npx-pLZ12. The plasmid was electroporated into GBS competent cells and transformants were selected by growth on 3 μg/ml chloramphenicol.

### RNA Isolation and RT-PCR

RNA isolation, cDNA synthesis and RT-PCR were performed as described previously (Korir et al., 2014; Korir, 2016). Briefly, RNA samples were collected from bacterial culture in liquid medium by adding two volumes of RNAprotect Bacteria Reagent (Qiagen) or from bacteria inside macrophages by washing the cells twice with PBS then adding 1 ml RNAprotect Bacteria Reagent directly to the cells. RNA was then extracted using the RNeasy minikit (Qiagen) using the “Enzymatic Lysis, Proteinase K Digestion, and Mechanical Disruption of Bacteria” protocol in the RNAprotect handbook. Residual genomic DNA was removed using the Turbo DNA-free kit (Ambion). The iScript Select cDNA synthesis kit was used to synthesize cDNA using random primers. As a control to test for DNA contamination, samples were processed without reverse transcriptase. RT-PCR analysis was performed using the iQ SYBR Supermix (Bio-Rad) and gene specific primers (Table 2). Relative fold change in gene expression was calculated using the 2^-ΔΔCt^ method and relative transcript level was calculated using 2^-ΔCt^ method; *gyrA* was used as the internal control for both (Schmittgen and Livak, 2008).

**Table 2:** Oligonucleotide primers used for RT-PCR.

Gene	Forward primer (5′ to 3′)	Reverse primer (5′ to 3′)
*gyrA*	CGGGACACGTACAGGCTACT	CGATACGAGAAGCTCCCACA
*npx*	GACCGCCTTCCCTGATTCAT	TAGCAGTTGTTGGGGCAGG
*ahpC*	GCGGATGTATTGAGCAGCAC	GATCCAGACGGTGTCATCCA
*tpx*	GCATTTGTTAACGCGTGCAG	CAGCATTAATCGCCGCTTCG


### H_2_O_2_ Quantification

Bacterial cultures were incubated at 37°C with the indicated concentrations of H_2_O_2_ in sodium phosphate buffer for 1 h in a 96 well plate. Buffer with bacteria alone (no H_2_O_2_ added) and buffer with H_2_O_2_ but without cultures, were included as controls. After 1 h, the bacteria were pelleted and supernatants were transferred to a new 96 well plate. The amount of H_2_O_2_ remaining in the supernatants was determined using the Fluorimetric Hydrogen Peroxide Assay Kit (Sigma) according to the manufacturer’s directions. The concentration of H_2_O_2_ was calculated by comparing fluorescence readings from the samples to a standard curve. Percent detoxification was calculated by normalizing data to the H_2_O_2_ controls without bacterial cultures.

### Survival in H_2_O_2_ and Multiple Stress Medium

The ability of GBS to survive phagosomal stress was described in our prior study (Korir et al., 2016). Briefly, GBS cultures were exposed to either 5 mM H_2_O_2_ alone, 5 mM H_2_O_2_ in sodium phosphate buffer at pH 4.5, or a multiple stress medium, which consisted of 1.5 mM H_2_O_2_, 3 mM NaNO_2_, 100 μM CuCl_2_, and 100 μg/ml lysozyme in acidic sodium phosphate buffer (pH 4.5) for 1 h then diluted and plated to enumerate viable bacteria. Percent survival was calculated by normalizing viable bacteria in the treated sample to the untreated samples.

### Human Placental Macrophage Isolation

Placental tissue was collected from non-laboring women who delivered healthy, full term infants by Caesarian section. De-identified tissue samples were provided by the Cooperative Human Tissue Network, which is funded by the National Cancer Institute. Placental macrophages were isolated according to our previously published methods (Tetz et al., 2015).

### Ethics Statement

This study was carried out in accordance with the recommendations of the Vanderbilt University Institutional Review Board. All placentae were obtained fully de-identified by the Cooperative Human Tissue Network. This protocol was approved by the Institutional Review Board (Approval No. 131607), which determined that our studies did not qualify as “human subject” research. The study did not involve interaction or intervention with a human subject and the specimens were stripped of identifiable information prior to the investigator receiving the samples.

### ROS Production in Placental Macrophages

The level of ROS produced by human placental macrophages after GBS infection was quantified as described in our prior study (Korir et al., 2016). Briefly, macrophages were labeled with 10 μM Carboxyl H2DCF-DA using the manufacturer’s recommendations (Invitrogen), washed and infected with GBS using a MOI of 50:1. ROS was also induced using 200 ng/mL PMA as a positive control, while ROS production was measured 30 min after infection and was normalized as percent of the untreated control. The 30 min time point was chosen based on data generated in our prior study (Korir et al., 2016) and to prevent the induction of a cytotoxic response.

### Statistical Analysis

GraphPad Prism version 5 was used for all statistical analyses. Comparisons across multiple groups or with two parameters were analyzed using one-way or two-way ANOVA respectively using Bonferroni post-tests where appropriate. Comparisons with *P* ≤ 0.05 were accepted as statistically significant.

## Results

### NADH Peroxidase Is Highly Upregulated in GBS During Intracellular Survival

**FIGURE 1:**
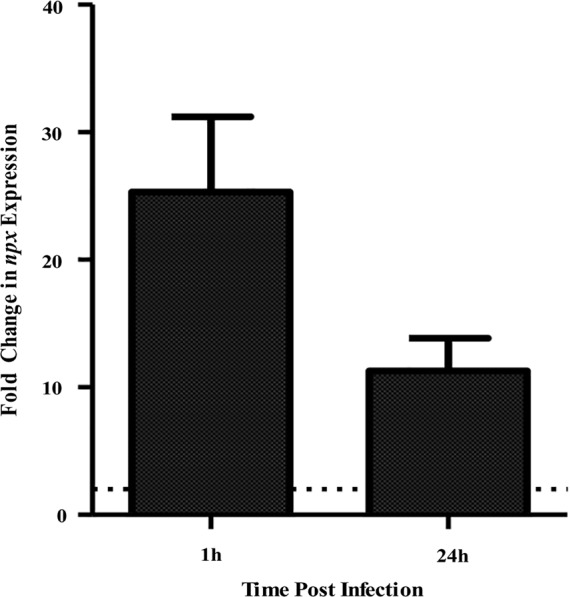
Expression of *npx* at 1 and 24 h survival inside PMA-treated human macrophages tested using qPCR. Fold change in expression was calculated relative to gene expression during growth in RPMI medium alone for 1 h. Dotted line indicates twofold change in gene expression. Data shown is average ± SD of three separate biological replicates.

In a previous study, we determined that a ST-17 isolate, GB00112, was capable of surviving inside human THP-1 macrophages significantly longer than a ST-12 isolate (Korir et al., 2016). This same strain was used for RNA sequencing (data not shown) to identify factors that are upregulated during intracellular survival relative to growth in medium alone (Korir, 2016). Significant upregulation of locus GB112_04315 (*npx*), which encodes a putative NADH peroxidase (Npx), was confirmed to be highly upregulated at both 1 and 24 h post-infection (Figure 1). Furthermore, a BLAST search of 252 published GBS genomes with full *npx* coverage showed a high (98.9–100% identity) level of homology across strains.

### The Role of Npx in H_2_O_2_ Survival

The presumed function of NADH peroxidases is to detoxify endogenously produced H_2_O_2_, such as that produced during aerobic growth, metabolism, or through dismutation of superoxide (La Carbona et al., 2007). Because they could also detoxify exogenous sources of H_2_O_2_, including those that occur during the ROS burst in macrophages, we sought to determine the role of Npx in survival against H_2_O_2_ stress. To do this, a deletion mutant was constructed in the ST-17, GB00112 strain background and complementation was performed *in trans* using the pLZ12 plasmid containing the constitutive *rofA* promoter (Neely et al., 2003) controlling the wild type (WT) allele of *npx*.

**FIGURE 2:**
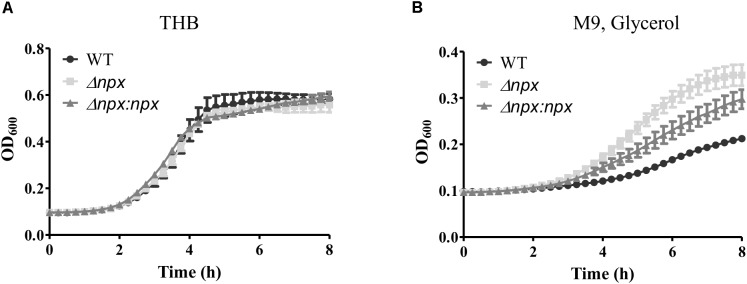
Growth of GBS utilizing different carbon sources. GBS strains were grown in either **(A)** THB, which contains glucose as the main carbon source or **(B)** M9 medium using glycerol as the sole carbon source. Cultures were grown in 96 well plates and OD_600_ was read every 15 min using Bio Tek Cytation 3 plate reader. Data shown is representative of three separate biological replicates. Error bars represent SD of six technical replicates.

To examine the ability of Δ*npx* to withstand high levels of endogenously produced H_2_O_2_, we compared the level of growth in complex medium (THB), which consists of glucose as the main carbon source, to that of growth in medium with glycerol as the sole carbon source. H_2_O_2_ is produced in lactic acid bacteria during glycerol metabolism through α-glycerophosphate oxidation (Whittenbury, 1964; Koditschek and Umbreit, 1969). Growth was very similar for all three strains when grown on glucose (Figure 2A); however, Δ*npx* showed a higher level of growth in glycerol compared to the WT strain. The Δ*npx*:*npx* strain had a level of growth that was between the mutant and WT indicating incomplete complementation under higher oxidative stress conditions (Figure 2B).

**FIGURE 3:**
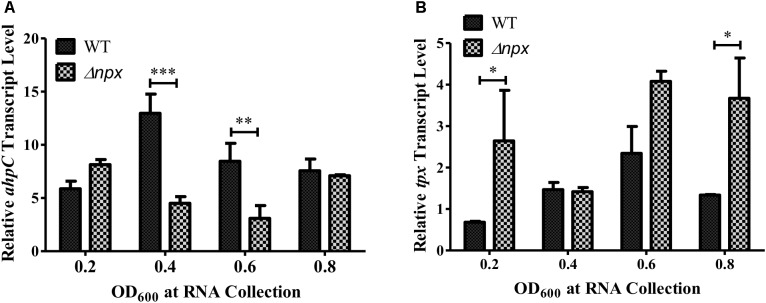
Peroxidase expression during growth in M9 with glycerol as the sole carbon source. RNA was collected at the indicated time points and relative transcript levels of **(A)** *ahpC* or **(B)** *tpx* was determined using *gyrA* as the internal control gene. Data shown is representative of two separate biological replicates. Error bars and statistical analysis shown were done using three technical replicates (^∗^*P* < 0.05, ^∗∗^*P* < 0.01, ^∗∗∗^*P* < 0.001).

The GBS genome also encodes two other putative peroxidases: a thiol peroxidase (*tpx*) and an alkylhydroperoxide reductase (*ahpC*), both of which could also contribute to ROS detoxification in the cell (Glaser et al., 2002). Therefore, we hypothesized that one or both of these genes could be overexpressed in the Δ*npx* strain to compensate for the loss of Npx. Indeed, such overexpression could allow for more rapid detoxification of the endogenously produced H_2_O_2_ during growth on glycerol, thereby resulting in a higher level of growth in the mutant. To test this hypothesis, we collected RNA at various stages of growth in medium containing glycerol as the sole carbon source and examined gene expression of *ahpC* and *tpx.* Since the strains exhibited different growth rates over time, samples were collected based on OD_600_ rather than time of growth to ensure cells were in the same growth phase at time of collection. Expression of *ahpC* was significantly lower in Δ*npx* compared to the WT around mid-log phase (OD_600_ 0.4 and 0.6) but was similar to WT expression at early and late log phase (Figure 3A). Expression of *tpx* was higher in Δ*npx* relative to WT levels at all times except OD_600_ 0.4, although the difference was not significant at OD_600_ 0.6 (Figure 3B). For each replicate performed, the fold-change value in gene expression varied slightly between replicates, however, the overall trends were the same. It is also important to note that the discrepancy between maximum OD_600_ in Figure 2B and OD_600_ values reported in Figure 3 are due to slightly different growth conditions. Cultures were grown in 96-well plates for Figure 2B, which results in a lower maximum OD_600_ compared to growth in tubes with much larger volumes for Figure 3. Despite the difference in maximum OD_600_ reached during culture, all strains exhibited similar growth patterns relative to the others (data not shown).

**FIGURE 4:**
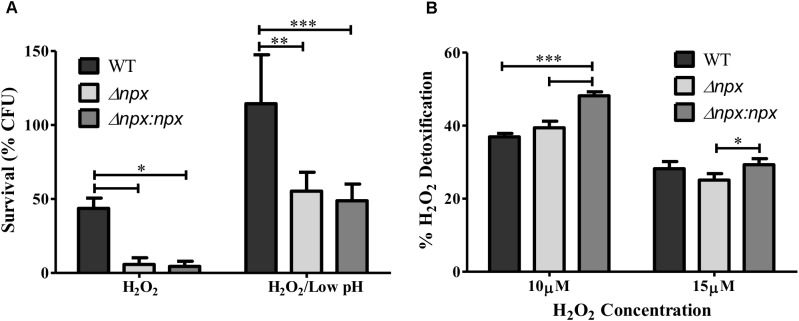
Role of the *npx* locus in response to H_2_O_2_. **(A)** Survival of GBS after 1 h exposure to 5 mM H_2_O_2_ alone or in combination with pH 4.5. Survival is expressed as the percent of CFU after exposure relative to the untreated control. **(B)** The level of H_2_O_2_ detoxification by GBS at two different H_2_O_2_ concentrations. Percent detoxification was calculated by normalizing the amount of H_2_O_2_ remaining after incubation with GBS to the amount of H_2_O_2_ in the culture free control. Data shown is average ± SD of three separate biological replicates (^∗^*P* < 0.05, ^∗∗^*P* < 0.01, ^∗∗∗^*P* < 0.001).

**FIGURE 5:**
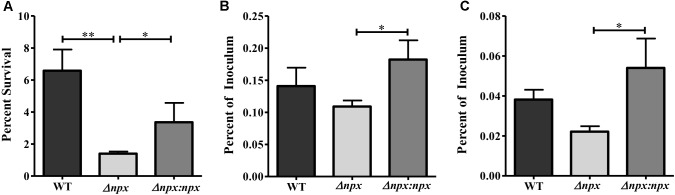
The role of the *npx* locus in surviving phagosomal stress. **(A)** Survival of GBS in a multiple stress medium consisting of acidic pH (pH 4.5), H_2_O_2_, NO, lysozyme, and Cu^2+^. Survival was calculated by normalizing the number of CFUs after treatment to the untreated control. **(B,C)** Survival of GBS inside PMA treated THP-1 human macrophages after 1 h **(B)** and 24 h **(C)**. The number of intracellular bacteria was normalized to the total amount of bacteria in the well after the initial infection period. Data shown is representative of three separate biological replicates. Error bars and statistical analysis shown were done using three technical replicates (^∗^*P* < 0.05, ^∗∗^*P* < 0.01).

**FIGURE 6:**
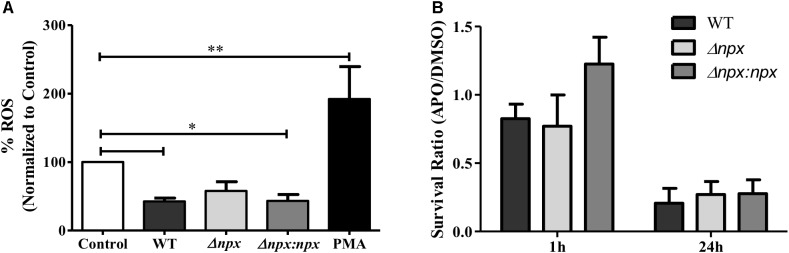
Association between *npx* and the ROS burst in macrophages. **(A)** ROS production in placental macrophages was measured 30 min after exposure to medium alone (control), GBS, or 200 ng/ml PMA as a positive control. ROS production was normalized to the untreated control. **(B)** Intracellular survival of GBS strains was determined at 1 and 24 h post-infection. Macrophages were treated with either DMSO (negative control) or apocynin (APO) 30 min prior to infection. Survival in APO is shown as a ratio relative to survival in DMSO. Data shown is average ± SD of three separate biological replicates (^∗^*P* < 0.05, ^∗∗^*P* < 0.01).

We next examined the ability of these strains to survive in the presence of an exogenous source of H_2_O_2_ by incubating the culture in 5 mM H_2_O_2_ and determining the level of survival after 1 h. The *npx* mutant was significantly more susceptible to killing in the presence of H_2_O_2_ relative to the WT (Figure 4A). Surprisingly, this phenotype was not complemented in Δ*npx*:*npx* despite this strain showing similar phenotypes to the WT strain in other experiments (Figures 5, 6). In our previous study, we reported that the ability of this ST-17 WT stain to survive H_2_O_2_ exposure was rescued when H_2_O_2_ stress was combined with low pH exposure (pH = 4.5) (Korir et al., 2016). Therefore, we next examined survival of these strains in the presence of both 5 mM H_2_O_2_ and pH 4.5. Consistent with our previous results, the survival of the WT was restored in the combined stress. Interestingly, both Δ*npx* and Δ*npx*:*npx* showed reduced survival in the combined stress relative to the WT; however, their survival rate was very similar to that of the WT in H_2_O_2_ alone (Figure 4A).

To further explore the mechanism of how Npx aids in survival against H_2_O_2_, we determined how well GBS was able to detoxify exogenous H_2_O_2_ by measuring the level of H_2_O_2_ present in the medium before and after incubation. No significant difference in H_2_O_2_ detoxification was detected between Δ*npx* and WT in any H_2_O_2_ concentration examined. However, the complemented *npx* mutant detoxified H_2_O_2_ at a significantly higher rate than Δ*npx* and WT at 10 μM H_2_O_2_ (Figure 4B).

### Contribution of Npx to GBS Survival in the Presence of Phagosomal Stressors

Since *npx* was initially found to be upregulated during macrophage survival, we next examined the role that Npx plays in the survival of GBS inside macrophages. We first examined the ability of these strains to survive in our previously developed multiple stress medium that consists of a number of stressors found in the phagosome, including acidic pH, H_2_O_2_, NO, Cu^2+^, and lysozyme (Korir et al., 2016). The Δ*npx* strain was more susceptible to killing by this medium relative to the WT, while this phenotype was partially rescued in the Δ*npx*:*npx* strain (Figure 5A). Next, the ability of these strains to survive inside PMA treated THP-1 human macrophages was determined at 1 h (Figure 5B) and 24 h (Figure 5C) post-infection. At both time points, Δ*npx* showed a slight, but insignificant decrease in ability to survive. Although the Δ*npx*:*npx* strain survived similarly when compared to the WT, it had a significantly enhanced ability to survive compared to Δ*npx*.

Because GBS is capable of inhibiting the ROS burst in macrophages (Korir et al., 2016), we examined ROS production by human placental macrophages 30 min post-infection with our GBS strains. Consistent with previous results, the WT significantly reduced ROS production relative to the untreated control as well as the positive control, indicating inhibition of the ROS burst. Although there was no significant difference between WT and Δ*npx*, the Δ*npx* strain did not significantly reduce ROS production relative to the negative control as observed in the WT. Importantly, Δ*npx* did not *induce* a ROS burst as was observed with the induced positive control, suggesting it was still capable of partially inhibiting the ROS burst. The WT phenotype was fully restored in the complemented strain (Figure 6A).

Finally, we sought to determine how the macrophage oxidative burst impacts intracellular survival by examining the ability of GBS to survive inside macrophages that were pretreated with either DMSO as a negative control or apocynin, an inhibitor of ROS production in phagocytic cells (Stolk et al., 1994). To highlight the specific impact of apocynin on intracellular survival, survival in apocynin treated macrophages was normalized to survival in control macrophages. At 1 h post-infection, apocynin treatment did not reduce survival of any strains relative to the DMSO negative control, while at 24 h post-infection, apocynin treatment reduced survival to approximately 25% of the DMSO negative control for all three strains. Overall, apocynin treatment had a similar impact on survival of all three strains (Figure 6B). It is important to note that no difference in survival was observed in the WT (*P* = 0.15), Δ*npx* (*P* = 0.30), or Δ*npx*:*npx* (*P* = 0.31) strains when grown in RPMI and 500 mM apocynin after 24 h compared to DMSO and performed in triplicate.

## Discussion

Macrophages play an important role in establishing immune tolerance during pregnancy as well as immune defense against invading pathogens. However, pathogens with the ability to survive inside these macrophages can evade immune system clearing and cross host cell barriers undetected inside these important immune cells. Therefore, it is essential to have a better understanding of how these pathogens remain inside macrophages in order to develop new therapeutics to eliminate this intracellular threat.

Here, as well as in previous studies, GBS has been shown to withstand high levels of oxidative stress as well as inhibit the ROS burst in macrophages (Poyart et al., 2001; Cumley et al., 2012; Korir et al., 2016). The ability of GBS to resist oxidative damage by the ROS burst is in part due to superoxide dismutase; however, GBS is catalase negative and therefore, requires other ways to detoxify H_2_O_2_. In this study we identified a putative NADH peroxidase to be highly upregulated during intracellular survival and demonstrated that *npx* contributes to the mechanism employed by GBS to withstand H_2_O_2_ as well as inhibiting the ROS burst. However, it is likely not the only factor contributing to H_2_O_2_ survival as the *npx* mutant showed a higher level of growth with endogenous H_2_O_2_ stress relative to the WT, was still able to detoxify H_2_O_2_ at levels similar to that of the WT, and was not completely killed by H_2_O_2_ stress or macrophages. The *npx* mutant also inhibited the ROS burst but not to the same extent as the WT. Indeed, a significant reduction in ROS relative to the uninduced control was observed in the WT but not in Δ*npx*. Genome sequencing shows that the GBS genome also encodes a thiol peroxidase (*tpx*) and an alkylhydroperoxide reductase (*ahpC*) that could contribute to H_2_O_2_ detoxification (Glaser et al., 2002), though the role of these two genes in ROS defense requires further investigation for their involvement in this mechanism. Our results, which were performed in duplicate and revealed similar expression trends by growth stage in glycerol, show that *tpx* was expressed at higher levels in Δ*npx* relative to the WT. Hence, *tpx* expression may be upregulated in the Δ*npx* background to compensate for the loss of Npx function during increased production of H_2_O_2_ through endogenous sources. This finding suggests that Tpx does indeed function to aid in ROS detoxification, however, further studies are required to draw any conclusions on the function of Tpx and confirm these differences. Additional studies are also needed to determine whether each strain varies in the level of phagocytic uptake.

We have shown that Npx plays a role in survival against exogenous sources of H_2_O_2_. However, the exact mechanism by which it does this has yet to be determined. NADH peroxidases have been shown to detoxify H_2_O_2_ by converting it to H_2_O in a NADH dependent manner; however, when we examined reduction of H_2_O_2_ in the presence of Δ*npx* vs. WT as a measure of detoxification, we observed no significant difference. This result could in part be due to the low concentration of H_2_O_2_ used this assay. At such low concentrations, it is possible that other enzymes present in GBS were able to detoxify the H_2_O_2_ and that Npx was not needed or induced in the WT. When higher concentrations were used in this assay, however, it was difficult to see reduced levels of H_2_O_2_ in the presence of WT GBS vs. the untreated control (data not shown). Future work will require the use of purified protein with H_2_O_2_ in kinetic assays similar to that done with purified NADH peroxidase from *Enterococcus faecalis* (Parsonage et al., 1993) in order to determine if Npx does indeed directly detoxify H_2_O_2_.

In a few of our experiments, we observed either lack of complementation or incomplete complementation despite seeing complementation in other experiments. Upon further examination of the genome sequence of the WT strain (GB00112), we identified a gene immediately downstream of *npx*, which could potentially be within the same operon as *npx*. This gene, GB112_04310, is annotated as a hypothetical protein. Because certain peroxidases require reductases in order to recycle the enzyme (Poole, 2005), it is possible that this hypothetical protein may serve as a reductase for Npx or play a role in activating the reductase. Although we designed our deletion mutant to limit polar effects as much as possible, there is a chance that deletion of *npx* negatively impacted GB112_04310 expression. In this case, it is possible that the plasmid-expressed Npx may not have been getting recycled for further enzymatic activity in our complemented strain. This hypothesis is supported by our finding that the Δ*npx* phenotypes without complementation were observed in conditions with higher concentrations of H_2_O_2_ such as during growth with glycerol as the sole carbon source or when high concentrations of H_2_O_2_ were directly added to the culture. Hence, it is possible that all available Npx was used before adequate amounts of the H_2_O_2_ was removed, and Npx was unable to be recycled to continue removing H_2_O_2_. Further exploration of this possible operon, the catalytic activity of Npx, and the function of GB112_04310, however, is required for confirmation.

Although *npx* was highly expressed during survival inside THP-1 macrophages, we did not observe a significant reduction in survival in the *npx* mutant relative to the WT. This discrepancy could be due, in part, to other peroxidases compensating for the loss of *npx* or the type of cells used. Although THP-1 cells have been used in a number of studies and there are many advantages of using cell lines, they do not replicate the conditions as well as primary cells or *in vivo* models would (Kaur and Dufour, 2012; Chanput et al., 2014). Future work will therefore need to examine this mutant using both primary cell lines that produce a robust ROS response and in animal models to determine how loss of *npx* impacts GBS virulence. Evaluating the role of this gene in different strain backgrounds will also be important, particularly given our finding that different strains vary in their ability to survive inside THP-1 cells (Korir et al., 2016).

A previous study that examined survival of GBS inside J774 murine macrophages found that apocynin treatement did not affect GBS survival through 9 h post-infection (Cumley et al., 2012). Consistent with this finding, we did not observe reduced survival at 1 h; however, a significant reduction in survival was observed at 24 h post-infection. The difference between our current findings and the previous study could be due to timing differences as we did not sample between 1 and 24 h, which could represent a limitation. Indeed, these two time points were chosen solely based on the methodology utilized and differences observed in our prior study (Korir et al., 2016). We had hypothesized that inhibiting the ROS burst would increase the ability of GBS to survive intracellularly, yet our unexpected results show a reduction in intracellular survival. This finding suggests that either: (1) the ROS burst or something influenced by the ROS burst is needed for long term survival in macrophages; or (2) the long term inhibition of ROS production is negatively impacting the macrophages themselves. Although we examined cell permeabilization as a measure of cell death, we did not observe a difference with apocynin (data not shown). It is therefore clear that the mechanism of ROS inhibition requires further investigation.

We previously demonstrated that the WT strain, GB00112 could inhibit ROS production during infection of human placental macrophages (Korir et al., 2016), yet the mechanism by which GBS does this in macrophages has yet to be determined. It could involve rapid detoxification of ROS as it is being produced, the inhibition of the NOX2 NADPH oxidase, or a combination of the two. In this study, we showed that the *npx* mutant strain was still capable of inhibiting ROS production in human placental macrophages, but to a lesser extent than that of both the WT and the complemented mutant; differences between the strains, however, were not statistically significant. Nonetheless, this finding suggests that, although Npx is not necessary for ROS inhibition, it is involved in full ROS inhibition. Because the proposed function of NADH peroxidases is to directly detoxify H_2_O_2_, the possible mechanism of ROS inhibition by Npx is in detoxification of the generated ROS. However, we were unable to observe differences in H_2_O_2_ detoxification abilities between the mutant and WT, and thus, it is difficult to deduce the exact role that Npx plays in ROS inhibition without further investigation.

In *E. faecalis*, the well-characterized *npr* gene, which also encodes a NADH peroxidase, has 23% homology to the GBS *npx* examined in this study. Congruent with the current findings, an *E. faecalis npr* mutant had similar growth to the WT under normal growth conditions, but was more susceptible to killing by H_2_O_2_ as well as macrophages (La Carbona et al., 2007). Since NADH peroxidases have been found in other Firmicutes members (Sakamoto and Komagata, 1996), they likely represent a conserved mechanism for survival in the presence of intracellular H_2_O_2_ and ROS stress.

In summary, this study defines Npx as a key factor that allows GBS to survive oxidative and phagosomal stress, and contributes to the inhibition of the ROS burst in human macrophages. Additional studies should focus on determining the exact mechanism by which Npx contributes to these important virulence traits and understanding the role that other genes play in withstanding oxidative stress as well as other stressors (e.g., nitric oxide). Understanding the mechanism that GBS uses to survive phagosomal stress remains largely unknown and requires further study in order to define new ways to treat and prevent GBS infections.

## Author Contributions

MK, JG, DA, and SM contributed to the concepts and design of the study. MK, RF, and LR performed the experiments. MK, RF, and SM analyzed the data. MK and SM drafted the manuscript. All authors contributed to and edited the final version of the manuscript.

## Conflict of Interest Statement

The authors declare that the research was conducted in the absence of any commercial or financial relationships that could be construed as a potential conflict of interest. The reviewer AB and handling Editor declared their shared affiliation at time of review.
